# The Shifting Demographic Landscape of Influenza

**DOI:** 10.1371/currents.RRN1047

**Published:** 2009-10-05

**Authors:** Shweta Bansal, Babak Pourbohloul, Nathaniel Hupert, Bryan Grenfell, Lauren Ancel Meyers

**Affiliations:** ^*^Center for Infectious Disease Dynamics, Penn State University; ^†^Division of Mathematical Modeling, British Columbia Centre for Disease Control; ^‡^Weill Cornell Medical College (NYC) and Preparedness Modeling Unit, U.S. Centers for Disease Control and Prevention (CDC, Atlanta); ^§^Princeton University and ^¶^The University of Texas at Austin

## Abstract

Background: As Pandemic (H1N1) 2009 influenza spreads around the globe, it strikes school-age children more often than adults. Although there is some evidence of pre-existing immunity among older adults, this alone may not explain the significant gap in age-specific infection rates.

Methods & Findings: Based on a retrospective analysis of pandemic strains of influenza from the last century, we show that school-age children typically experience the highest attack rates in primarily naive populations, with the burden shifting to adults during the subsequent season. Using a parsimonious network-based mathematical model which incorporates the changing distribution of contacts in the susceptible population, we demonstrate that new pandemic strains of influenza are expected to shift the epidemiological landscape in exactly this way.

Conclusions: Our results provide a simple demographic explanation for the age bias observed for H1N1/09 attack rates, and a prediction that this bias will shift in coming months. These results also have significant implications for the allocation of public health resources including vaccine distribution policies.

**Introduction**

In March 2009, a new A/H1N1 influenza strain (Pandemic (H1N1) 2009 influenza or H1N1/09) emerged in humans in Mexico and by early June 2009,the World Health Organization (WHO) had raised the worldwide pandemic alert level to signal a global pandemic of a novel influenza virus. Since the WHO declaration of a pandemic, the new H1N1/09 virus has continued spread across the globe, causing growing outbreaks in most countries [1]. Public health officials anticipate that there will be more H1N1/09 cases, hospitalizations and deaths throughout the fall and winter in the northern hemisphere [2]. Early epidemiological data suggest that the 2008-2009 seasonal influenza vaccines are not effective against the new strain but that older individuals who were previously infected by another H1N1 strain may be less likely to develop clinical infection [3]. Multiple manufacturers are now developing vaccines for H1N1/09, and as a best case scenario, the distribution may begin as early as October 2009, until which time the virus will continue to spread.

Influenza is a complex and continually changing disease that infects individuals of all ages. In contrast to diseases like measles and rubella, the dynamics of influenza are strongly influenced by the evolution of immunological properties of the pathogen [4]. The epidemiological landscape of flu is dynamically shaped by cycles of naturally-acquired immunity (through infection) and immune escape (through viral evolution). Based on data from prior influenza pandemics and a simple network-based mathematical model, we argue that, for a newly introduced strain of influenza, this process will cause a shift in the demographic burden of influenza from children to adults. Our analysis also has implications for optimal vaccine allocation. We echo our previous conclusions that high-risk groups should receive highest priority and direct protection when vaccine supplies are limited [5]. However, secondary efforts should focus on indirect protection of groups with greatest potential for infection, whose identities may change over time. 

**Methods**

Population Model

Influenza spreads during close contacts between susceptible and infected individuals. The likelihood of a person becoming exposed to disease will strongly depend on the number and intensity of his or her interactions [6] [7] [8] [9] [10]. To study how the interplay of heterogeneities in interaction patterns and infection-induced immunity drive the demographic progression of pandemic influenza, we use network models in which individuals are represented as *nodes*, and *edges* connecting nodes represent disease-causing interactions, or *contacts*, which may occur between individuals during an infectious period. The number of edges for a given node is known as the node's degree, and the probability distribution of degrees over all nodes is referred to as the *degree distribution*. 

Our network model represents an urban area population and is based on data for the city of Vancouver, British Columbia [7]. We model the interaction patterns relevant for the spread of influenza in the population via a data-driven, activity-based contact network model. Each person is assigned an age based on census data from the city of Vancouver, British Columbia, and age-appropriate activities (e.g. school, work, nursing home, etc.) Contacts among individuals reflect household size, employment, school and hospital data also from Vancouver. (More details can be found in [5] [7].)The emergent age-specific contact patterns of our model closely resemble other empirical estimates (Figure 1), and parsimoniously incorporate individual-level heterogeneity. 

Modeling Immunity and Second Season Dynamics

We assume that an infected node will infect a susceptible contact with a given probability (known as *transmissibility*) that depends on both the infectiousness and susceptibility of the nodes. Once infected, a node cannot be reinfected during the same outbreak and will have resistance to infection during the subsequent season (Figure 2). The cross-immunity in the second season is assumed to be partial and we use α to represent the loss of immunity from one season to the next ( α = 0 is full immunity against future infection, α = 1 is complete loss of immunity, and intermediate values correspond to partial immunity). For influenza A, natural immunity acquired in one epidemic tends to be heterotypic for the second epidemic appearance of the virus, though several studies have shown that individuals infected by influenza A can be reinfected by antigenically similar strains during the following seasons. An estimated 8% of individuals who were infected in the 1918-1919 Spanish pandemic were reinfected in January-February 1920 [11]; and the relative risk for clinical illness during the second wave of the 1918 pandemic after infection in the first wave was estimated to be as low as 6% in U.S. Army personnel camps (but was found to be as high as 51% in one of the camps) [12]. Similar rates of reinfection have been estimated for the 1968 Hong Kong influenza pandemic [13] [14]. Generally, immunity loss for influenza A has been estimated at 5% per year [15], and an estimated 7.4% of previously infected individuals become fully susceptible within one year [16]. Should H1N1/09 produce a second season of transmission, these studies of prior pandemic strains suggest that . will likely lie somewhere between 0.05 and 0.10. Following infection, antibodies that recognize influenza surface antigens, hemagglutinin and neuraminidase, persist and are associated with resistance to reinfection [17]. There is evidence that anti-hemagglutinin (HA) antibodies limit reinfection by antigenically similar strains of influenza [18] [19] and anti-neuraminidase (NA) antibodies significantly reduce virus replication and release if reinfected [20] [21]. Thus, our model assumes that cross-immunity reduces both susceptibility and infectiousness by a factor .α. 

Most empirical studies measuring immunity to influenza measure a reduction in infection rate at the population scale. Thus, we model the spread of influenza in a partially immune population assuming perfect partial immunity, using empirical data for infection-acquired immunity to influenza described above. Perfect partial immunity implies that for a level of loss of partial immunity α, a proportion \begin{equation*}\left( 1-\alpha \right)\end{equation*} of the previously infected population is completely protected, while the remaining α are fully susceptible. (If empirical data on leaky partial immunity for influenza is available, the network-based leaky immunity model described in [22] can be used instead.) 

To consider disease dynamics beyond the initial pandemic period, we have developed a mathematical approach based on percolation methods. The standard bond percolation model [23] assumes no pre-existing immunity, thus is an appropriate model for the spread of a novel influenza strain. Using this method to model the spread of influenza in a naive population, we can define a residual network, which is the relevant contact network for a subsequent outbreak caused by the same pathogen in the same population. The residual network is made up of individuals who were not infected in the initial epidemic, a proportion . of who were infected but have lost immunity since infection and the edges joining them. We describe the residual network via its degree distribution, \begin{equation*}p_{res}\left( k_r \right)\end{equation*}, or the probability that a (susceptible) individual in the residual network has \begin{equation*}k_r\end{equation*} contacts with other (susceptible) individuals in the residual network (which we derive in Supplementary Information) [22]. Given the transmissibility of the second season pathogen, \begin{equation*}T_2\end{equation*} , (which may or may not be different than the transmissibility of the .first season pathogen),), and use bond percolation techniques to predict the consequences of a second season spread of infection. The relationship between transmissibility \begin{equation*}\left( T_1 \right)\end{equation*} and the reproductive number in a naive population \begin{equation*}\left( R_0 \right)\end{equation*} is described by:

\begin{equation*}R_{0}=T_{1}\frac{\langle k (k-1)\rangle}{\langle k\rangle}=T_{1}\frac{\sum_{k}k (k-1) p(k) }{\sum_{k}kp(k)}\end{equation*}

where \begin{equation*}p(k)\end{equation*} is the degree distribution in the naive population. Similarly, the effective reproductive number in the partially immune population \begin{equation*}(R_e)\end{equation*} is described by

\begin{equation*}R_{e}=T_{2}\frac{\langle k_{r}(k_{r}-1)\rangle}{\langle k_{r}\rangle}=T_{2}\frac{\sum_{k}k(k-1)p_{res}(k)}{\sum_{k}kp_{res}(k)}\end{equation*}

An individual will be susceptible to infection in the second season if they were not infected in the first season or if they were infected and have lost immunity (with probability α.). Thus, the probability that a node of degree \begin{equation*}k\end{equation*} is susceptible to infection in the second season is equal to:

\begin{equation*}S(k) = ( 1-T_1+T_1u_1 )^k + \alpha( 1-( 1-T_1+T_1u_1 )^k )\end{equation*}

where, \begin{equation*}T_1\end{equation*} is the transmissibility of the .first season influenza strain and \begin{equation*}u_1\end{equation*} is a quantity that can be calculated from bond percolation techniques and depends on both the population's contact structure as well as the transmissibility of the pathogen [23]. Also, the probability of infection in the second season to an individual of residual degree \begin{equation*}k_r\end{equation*} (number of edges in the residual network) can be computed as \begin{equation*}(1-(1-T_2+T_2u_2)^{k_r})\end{equation*}, where \begin{equation*}T_2\end{equation*} is the transmissibility of the second season influenza strain, and \begin{equation*}u_2\end{equation*} is a quantity that can be calculated from bond percolation techniques. We can combine these two quantities to .find the risk of infection to a node of (original) degree k in the second season:

\begin{equation*}R(k)=S(k)\sum_{k_r}^{}{p_{res}(k_r|k)(1-(1-T_2+T_2u_2)^{k_r})}\end{equation*}

where, \begin{equation*}p_{res}(k_r|k)\end{equation*} is the probability that a node will have residual degree \begin{equation*}k_r\end{equation*}, given that it has a degree of \begin{equation*}k\end{equation*} before the first season (and is derived in Supplementary Information) [22]. The values for risk of infection for the second season shown in Figure 3 were calculated using the above formulation, and verified by comparison to stochastic simulations (not shown). Stochastic simulations for this verification and Figure 3(A) assumed a simple percolation process with \begin{equation*}T_1\end{equation*} and \begin{equation*}T_2\end{equation*} as described.

Vaccination Priorities

We modeled vaccination priorities by randomly selecting individuals within the priority group (e.g. school-age children). We modeled cross-immunity from exposure to pre-1957 H1N1 strains of influenza by immunizing a randomly chosen subset of adults (9%) and elderly (33%). 

**Results**

Evidence for a Fluctuating Landscape

Pandemic influenza is feared for its severe excess mortality [24], rates for which can vary greatly. For most seasonal and pandemic flu, the elderly and very young are at highest risk for severe disease; however, the 1918 Spanish flu pandemic is believed to have been deadliest for 20-40 year olds [25]. influenza morbidity and attack rates also vary among demographic groups. Epidemiological studies and conventional wisdom suggest that school children have the highest attack rates and ultimately fuel transmission throughout the community [26] [27] [28] [29] [30]. Data from the three known influenza emergence events in the twentieth century initially show this bias towards school-aged children (Figure 3). When we look beyond the initial pandemic period, however, the age-specific attack rates reverse, with the probability of infection in adults exceeding that of children.

Data from H1N1/09 outbreaks thus far reveals a similar initial discrepancy in attack rates (Figure 3, [31] [32]). There is mounting evidence that cross-immunity from exposure to prior strains may be protecting older adults [3], as has been suggested for infection with the 1918 influenza strain [33]. However, there is a simple and complementary explanation for the differences in attack rates and subsequent age shifts that is based on the heterogeneous contact patterns underlying the spread of influenza.

Several diverse studies have estimated the distribution of contact patterns among age groups, primarily in urban populations [34] [35] [36] [37] [38]. Although the studies use different definitions of contact and contact rate, all but one suggest that children have the highest numbers of contacts followed by adults (Figure 1). Basic epidemiological theory suggests that, in the absence of intervention and cross-immunity, children should therefore have the highest attack rates [7] [39] [40]. As infection-induced immunity accumulates among the highly connected individuals, however, the infection cascades into other parts of the population [22] [41] [42]. In fact, stochastic simulations of disease transmission illustrate that even within a single influenza outbreak, the burden of disease shifts from children to adults as disease progresses from the most connected to more moderately connected portions of the population (Figure 4(A)).

Using our mathematical model, we calculate the expected age-specific attack rates in the .first and second seasons given the contact structure of the network (its degree distribution), the infectiousness of the strain, and the level of partial immunity from one season to the next. We .find that the attack rate shifts shown in Figure 3 are a natural outcome of the contact patterns described in Figure 1. Intuitively, the likelihood of becoming infected during the initial phase of the pandemic increases with number of contacts. However, if the strain makes a second appearance, then the relationship between contact patterns and epidemiological risk is altered by immunity acquired during the initial outbreak (Figure 4(B)). When the population is fully susceptible, the highest-degree nodes are most at risk for infection; and thus are likely to be protected against reinfection. In a partially immune population, while individuals with very few contacts maintain low levels of risk, moderately connected individuals become the most vulnerable subset of the population. This transition is expected to be more pronounced if high levels of immunity are maintained by individuals infected during the initial outbreak (Figure 4(B)).

If the reproductive number in the.first season is \begin{equation*}R_0=1.6\end{equation*} (as has been estimated for H1N1/09 [31] [43]), our model suggests that the returning strain will only invade if it is more transmissible (higher probability of transmission per contact) than the original strain; however, because of a reduced number of contacts among susceptible individuals, its effective reproductive number may be considerably lower than the original strain (Supplementary Information). Higher transmissibility can occur if the pathogen evolves to be more infectious or virulent [44] [45] or if the social structure changes to enhance transmission, for example, with the commencement of school or relaxation of social distancing measures. Children tend to have higher numbers of contacts than adults (Figure 4(C)) (who combined make up more than 80% of the population in most developed nations). Thus Figure 4 suggests that the burden of disease is expected to shift from school-age children to adults both during the initial pandemic and between the initial pandemic and the subsequent season, which is consistent with the patterns observed during the three influenza pandemics of the twentieth century (Figure 3).

Implications for Vaccination

The vaccination of school-age children has been suggested as an effective influenza control strategy [28] [46] [47] [48]; and school-age children are among the U.S. CDC's H1N1/09 vaccination priority groups [49]. Since school children are thought to be critical transmitters of flu, immunizing them can break potential chains of transmission before they reach the greater community. This strategy, however, hinges on the primacy of school-age children in influenza transmission and the general idea that the likelihood of catching and spreading flu is proportional to one's number of contacts [5] [50] [51] [52]. However, our study illustrates that naturally-acquired immunity may restructure the population so that the most highly connected individuals are no longer the most vulnerable nor the most likely to transmit infection. Thus the optimal vaccination strategy may depend on the recent epidemiological history of the population. For example, we consider a scenario in which there is a limited supply of vaccine doses (15% coverage) and consider two strategies: (i) vaccinate a random subset of children or (ii) vaccinate a random subset of adults (Figure 5). In other words, vaccinating the most highly connected age group or a more moderately connected age group. This analysis is meant to simply explore whether vaccination strategies to minimize transmission should shift as the disease alters the immunological structure the host population; and we do not consider other outcome measures such as mortality, years of life lost or economic costs [5] [53] [54].

Vaccination reduces the size of the epidemic through both direct protection of 15% of the population and indirect protection of others through partial herd immunity. Figure 5(A) shows that during the initial phase of pandemic spread, when the population is fully susceptible, it is more effective to vaccinate children than adults. This prediction reverses for a second season, with adult vaccination more effectively reducing total cases than school-age vaccination. We further consider the impact of resistance from exposure to prior strains of the same subtype among older adults (Figure 5(B)). In particular, there are estimates that 9% of adults and 33% of elderly are resistant to H1N1/09 from H1N1 infections prior to 1957 [3]. Even with historical cross-immunity, adult vaccination is expected to be more effective than school-aged vaccination in the second season. 

**Discussion**

Influenza transmission is constrained by contact patterns, which are influenced by individual behavior and sociological events. For example, the early transmission of H1N1/09 in Mexico City was likely hampered by the closing of schools for the two-week Holy Week period and the subsequent implementation of social distancing interventions including school closures [43]. These events prevented contacts that typically take place within schools that are thought to be pivotal to spread of flu through communities [47] [48]. The reverse is also true: the dynamics of infectious diseases can dramatically alter the structure of a host population. Outbreaks of fully immunizing diseases like measles permanently remove cases from the susceptible fraction of the population. influenza, along with many other partially immunizing diseases such as RSV, pertussis and rotavirus, provides temporary incomplete immunity. Individuals fade in and out of the epidemiological active portion of the population as they become infected and slowly regain susceptibility to future infection. When a novel influenza strain emerges into a pandemic, it works its way through the population, preferentially infecting and thus immunizing individuals with high numbers of contacts. It essentially prunes the underlying contact network by removing highly connected individuals and all of their connections. If the strain reemerges in the following season, it faces much sparser chains of susceptible individuals, in which spread is more limited and new groups are expected to bear the brunt of the epidemic. Our simple network-based mathematical model elucidates this phenomenon by both incorporating the heterogeneous distribution of contacts among age groups and tracking the changing immunological structure of the population from one season to the next [10] [22].

This model does not consider demographic processes such as births, deaths, and aging. We have found, however, that population aging has minimal impact on network structure or disease dynamics across levels of immunity (Supplementary Information). We also have not addressed the dynamics beyond two seasons and believe that, while the relative risks will continue to change, we cannot simply extrapolate our results to future seasons. When schools are in session, school children tend to have the highest numbers of contacts among all age groups [34] [55]. Consequently, they often form the leading edge of a pandemic [31] [56] [57]. Adults tend to have lower numbers of contacts and thus lower risk of infection, although they play an important role in spatially dispersing infection [58]. Based on estimated contact patterns, our network model suggests that attack rates for a novel strain of influenza should initially be biased towards children and then shift towards adults. This is consistent with estimated attack rates for the three major pandemics of the 20th century and early H1N1/09 epidemiological data.

This analysis suggests that we might experience a shift in H1N1/09 age-specific infection risks (and thus potential for infecting others) over the next 12 to 24 months, and that the optimal distribution of vaccines and other public health resources may change throughout this period. While many developed nations are aiming for universal vaccination, estimates for the delivery of vaccines range from late September through January 2010 [59] [60] [61]. As H1N1/09 continues to spread, it is changing the immunological landscape of the population. The most vulnerable groups in September 2009 may look very different than the those in November 2009. For example, if mass school-based transmission of H1N1/09 has occurred prior to the availability of vaccines, then perhaps school-aged children should receive lower priority than other groups and school closures should be de-emphasized as a disease control measure.

Although our study does not explicitly consider the important option of prioritizing groups at high risk for mortality, we echo our previous claim ([5]) that high-risk groups and critical personnel should receive highest priority when vaccine supplies are limited. Secondary efforts should focus on groups with greatest potential for becoming infected and infecting others in order to maximize indirect protection. This study suggests that the identities of these high transmission groups depend on the epidemiological history of the population. For seasonal influenza, the high risk age groups (elderly and infants) are distinct from the high transmission age group (school-age children). However, during the second season following a pandemic these priorities may align. In prior pandemics, the highest-risk age groups were young healthy adults (1918) or elderly and infants (1957 and 1968) [62]. Thus, in a partially immune population, prioritizing adults, elderly and infants may not only provide indirect protection by achieving the greatest herd Immunity but also directly protect those at risk of complications or death. 

**Figures**

**Figure:**
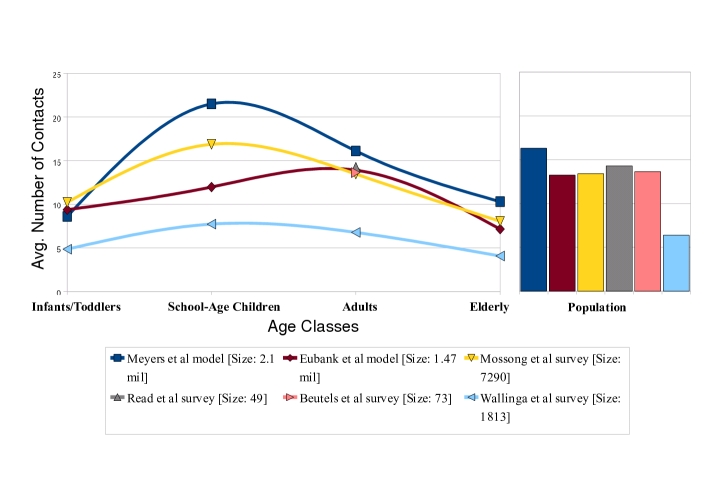
Figure 1 Estimated age-specific contact rates in an urban population: We compare six estimates for the mean degree by age of individuals (left panel) and the mean degree across the population (right panel). Meyers et al. [7] and Eubank et al. [38]are model-based estimates in which survey, census and other data were used to construct detailed computer simulations of contact patterns in Vancouver, BC and Portland, OR, respectively. The remaining four sets of estimates are inferred from responses to survey questions about the frequencies of (a) two-way conversations lasting three or more words in the physical presence of another individual, and (b) a physical contacts which involve skin-to-skin contact. The Wallinga study [37]includes only conversational contacts, while the Mossong, Read and Beutels studies [34] [35] [36] include both contact types. The Read and Beutels studies only include adults. Our model (based on [7]) measures contacts during an average infectious period, while the remaining studies measure daily contacts.

**Figure:**
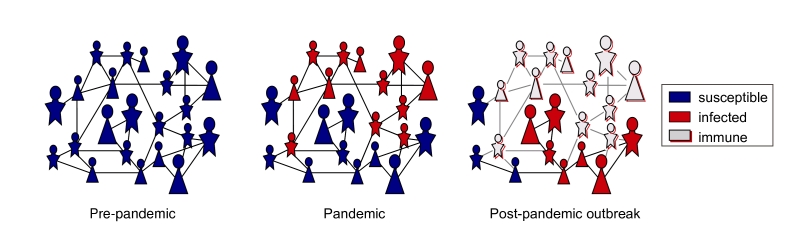
Figure 2 Changing immunological structure of a population throughout an influenza pandemic: Lines in these network diagrams indicate contacts through which influenza can spread. Prior to the introduction of a novel pandemic strain, most of the population is susceptible. The pandemic initially sweeps through the most connected portions of the populations, including groups of school-age children, leaving a wake of temporarily immunized individuals. The remaining susceptible population will consist of less connected portions of the population.

**Figure:**
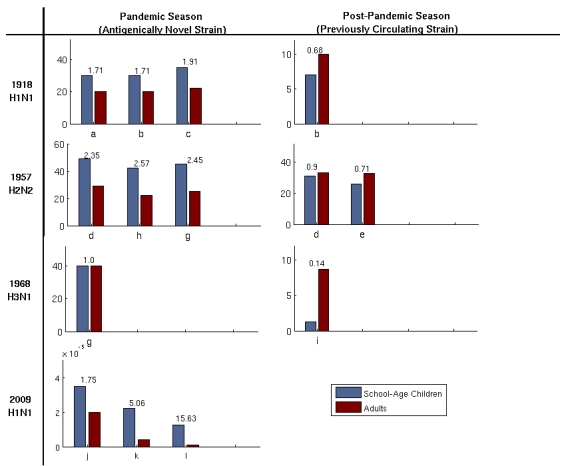
**Figure 3 Attack rates among adults and children during influenza pandemics and subsequent seasons: Multiple bars for a single strain represent data from different populations. Data are from a: [57], b: [56],c: [63], d: [64], e: [65], f: [66], g: [67], h: [68], i: [69], j: [70], k: [71], l: [32]. Numbers above bars represent odds ratios. While there are consistent qualitative patterns, the estimates are based on diverse data and methodologies and thus should not be compared quantitatively across studies. The 1968 Hong Kong H3N2 pandemic is the only one of the four strains that does not appear to have an initial bias towards children, which may be influenced by cross immunity from prior H2N2 infections as the two viruses shared nearly identical neuraminidase molecules [72]. Data for H1N1/09 is reported as number of confirmed cases as a proportion of age group size in the respective country.**

**Figure:**
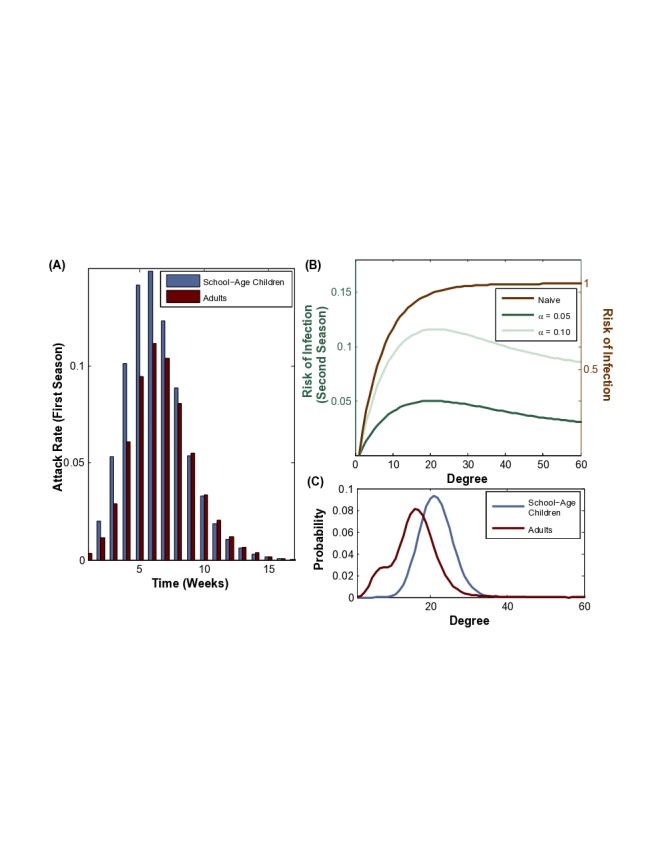
**Figure 4 Individual risk of influenza infection during two sequential outbreaks: (A) During the initial pandemic season, we notice a shift in the attack rate (the number of new cases during a week in an age group divided by the size of the age group). The attack rate among children is initially higher than the attack rate among adults, but this reverses after the epidemic peak. (B) During the initial pandemic, all individuals are susceptible, and risk of infection (defined in Methods) increases with number of contacts (dashed brown line, and right y-axis). During a subsequent outbreak the epidemiological risk landscape shifts towards moderately connected individuals, depending on the the level of immunity (green lines, and left y-axis) for  T1 = 0:09 (R0 = 1:6) and T2 = 0:15 (Re = 1:05; 1:16). (C) The degree distributions for school-age children (mean degree of 21.5) and adults (mean degree of 16.1) in our urban population network model. The bimodal adult degree distribution reflects heterogeneities in adult employment status.**

**Figure:**
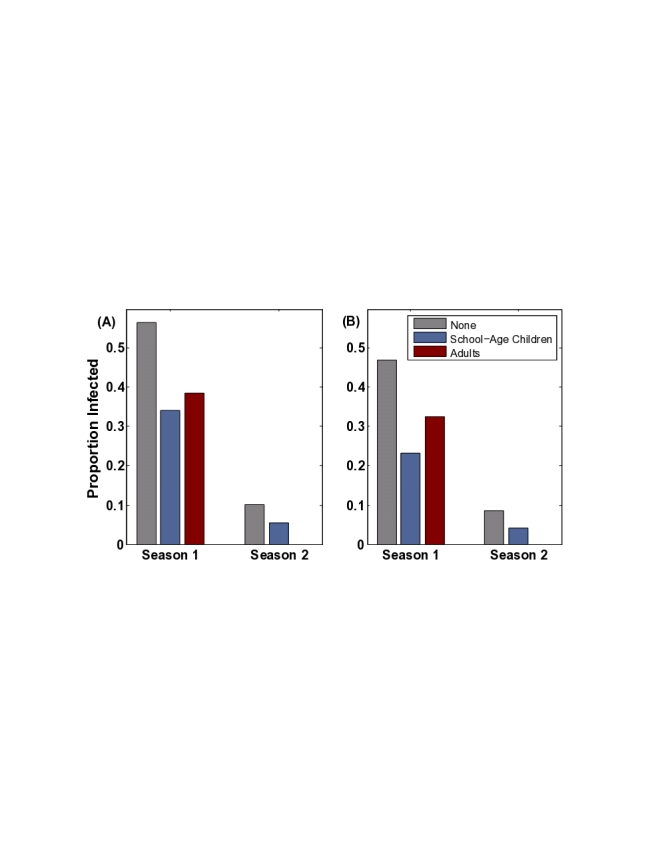
**Figure 5 Comparison of vaccination policies: (A) The impact of school-aged and adult vaccination priorities at 15% vaccine coverage in a naive (``Season 1”) and partially immune population (``Season 2”) population at alpha = 0.05 (B) The impact of these policies assuming pre-existing resistance among adults (9%) and elderly (33%) acquired through exposure to a strain of the same subtype prior to 1956. The first season pathogen has a reproductive ratio of R_0 = 1.6 and the second season pathogen has an effective reproductive ratio of R_e = 1.005**

**** **** **Acknowledgments**The authors thank the U.S. Centers for Disease Control and Prevention (CDC) Epidemiology/Surveillance Team for sharing aggregate case count data on the H1N1 outbreak in the United States; , and Joel Mossong, Jonathan Read, Phillipe Beutels, and Clement Tuberlin for sharing data; Jessica Metcalf for assistance with data; and Tom Hladish for discussions. **Funding Information**This work was supported by the RAPIDD program of the Science & Technology Directorate, Department of Homeland Security, and the Fogarty International Center, National Institutes of Health; grants from the James F. McDonnell Foundation, National Science Foundation (DEB-0749097), and NIH Models of Infectious Disease Agent Study (MIDAS) (U01-GM087719-01) to L.A.M; and support from the Canadian Institutes of Health Research (PTL97125 and PAP93425) and the Michael Smith Foundation for Health Research to B.P.**Competing Interests**The authors have declared that no competing resources exist.
